# What is end-stage knee osteoarthritis? A scoping and narrative review

**DOI:** 10.1016/j.ocarto.2026.100808

**Published:** 2026-04-30

**Authors:** Thorlene Egerton, Penny K. Campbell

**Affiliations:** Centre for Health, Exercise and Sports Medicine, The University of Melbourne, Parkville, Australia

**Keywords:** Osteoarthritis, Scoping review, Narrative review, Knee osteoarthritis, End-stage osteoarthritis

## Abstract

**Background:**

The term ‘end-stage knee osteoarthritis (OA)’ was explored with combined scoping and narrative review methodology to identify and critique definitions and current practice usage.

**Methods:**

Medline and 16 relevant websites were searched for the term ‘end-stage (knee) osteoarthritis’. Extracted text-based data were analysed with an inductive qualitative content analysis method and discussed in relation to contemporary understanding of knee OA.

**Results:**

From 130 resources, the term was almost universally used to mean *eligible for surgery*. Analysis of the 20 definitions identified the themes: (1) *Radiographic evidence approach* (based on joint imaging), (2) *Clinical criteria or symptom burden approach* (incorporated symptom and function experiences), (3) *Surgical indication* (term used synonymously to mean surgery is needed), and (4) *Integrated radiographic and clinical algorithm* (radiographic and clinical dimensions combined in a decision rule). Total knee arthroplasty was frequently reported as the ‘gold standard’ treatment. Pain and function can improve in people with end-stage OA and non-surgical treatment options are available.

**Critique and conclusion:**

Our review identified limited definitions. The term is often used in a way that is inconsistent with actual patient experiences. That is, ‘end-stage’ is not necessarily the ‘end’. Our recommendations include using different terminology in clinical practice (e.g., using ‘surgical threshold’ to mean when the person becomes eligible to consider surgery), and recognising the right time for surgery is complex and individual given the multidimensional and variable nature of the OA experience. For determining research study eligibility, we recommend using specific tools to measure clearly defined constructs.

## 1 Introduction

Osteoarthritis (OA) is a prevalent disease affecting over 600 million people worldwide in 2021, with the knee being the most commonly affected joint [1,2]. Some patients experience worsening of symptoms and/or structure, and may at some point be told they have reached what is commonly referred to as ‘end-stage knee OA’. This concept is widely used in clinical practice and research, yet it lacks a standardised definition and clear characterisation. Ambiguity in its meaning could lead to inconsistencies with patient care if clinicians interpret ‘end-stage’ differently, potentially leading to varied treatment recommendations. Inconsistent use of the term in research literature can complicate the interpretation of findings and comparisons between study results. In addition, the lack of clear criteria for end-stage knee OA may affect the timing and appropriateness of interventions, particularly regarding the decision for joint replacement surgery.

This review uses both scoping review and narrative review methods. We firstly explore how the term end-stage knee OA is defined in the literature and how it is used to inform or justify clinical practice. We then discuss and critique our findings with a goal of providing recommendations for practice and future research. Our specific objectives were to:

1. Identify and thematically synthesise the definitions of end-stage knee OA used in a sample of relevant literature.
2. Review how the concept of end-stage knee OA is applied in clinical contexts and decision-making processes according to the sample of literature.
3. Discuss and critique the definitions and clinical usage in the context of contemporary understanding of knee OA with the aim of providing conceptual clarity and recommendations for clinical practice and research.

## 2 Methods

Scoping review methods are used to systematically synthesise the research on a given topic in order to map the research or, as in this case, summarise the topic with the aim of providing conceptual clarity [[3], [4], [5]]. The PCC framework was operationalised as end-stage knee OA (concept) referenced in medical research and evidence-based literature (context) involving adults with knee OA (population). Narrative reviews provide an opportunity to critically interpret findings and propose new approaches based on evidence [4,6,7]. This hybrid scoping–narrative approach combines systematically summarising the findings with critical interpretation to generate deeper understanding [7,8]. The protocol was registered on August 27, 2025 (https://doi.org/10.17605/OSF.IO/WX6FV).

### 2.1 Search strategy

We developed a search strategy to balance transparency and reproducibility, with remaining pragmatic about likely scope and resources. An online search of Medline (OVID) was conducted on August 18, 2025 (Appendix A). An exhaustive retrieval of all mentions of the term would have been impractical and unnecessary to meet the aim of exploring the range of definitions and uses. Therefore, a purposeful sampling strategy with pre-specified limits was developed with the goal of including at least 100 articles. The search was limited by using a date limit (last 10 years) and study type filter (reviews, guidelines, trials, observational studies). The term ‘end-stage (knee) OA’ and variations in spelling of this term were searched. The search was restricted to studies focused on the knee, published in English and relating to humans. In addition, key OA websites and clinical practice guidelines were also searched. These sources were identified using a generative artificial intelligence tool (GPT-5) (Prompt: “Generate a list of English-language websites for osteoarthritis organisations”), the reviewers’ knowledge of reputable organisations, and two recent reviews of knee OA guidelines [9,10] (Appendix A).

### 2.2 Screening and extraction

Due to our search strategy, we expected all articles to pass title/abstract screening. Articles and documents were therefore imported directly into the full-text screening stage of Covidence systematic review software (Covidence, Veritas Health Innovation, Melbourne, Australia. https://www.covidence.org). Initially, twenty percent (25 articles) were screened by two reviewers for eligibility (included the term end-stage OA anywhere in the article, included the knee, humans, English), with a plan to screen the remaining articles for inclusion by one reviewer if conflicts were resolved with full agreement after discussion. Similarly, extraction was initially undertaken by both reviewers and checked for consistency after each group of ten articles. Extraction used a template within Covidence that was pilot tested prior to use. Once reviewers consistently agreed, the remainder were extracted by one reviewer. The following data were extracted: source (author, year, country); study design/document type; study aim(s); if the term end-stage knee OA was defined, explained, described or discussed; how was the term used in relation to clinical practice.

### 2.3 Data analysis

Descriptive statistics were collated from the extracted data, and qualitative thematic analysis [11] was used to synthesise how end-stage knee OA was defined, discussed and used in the literature. The thematic analysis was inductive and descriptive, without a predefined conceptual framework. Two reviewers (TE, a physiotherapist, and PC, a research trial coordinator, both with qualitative research experience) independently grouped comments under separately created original codes relating to the research question. The reviewers then discussed proposed codes and agreed on the themes.

### 2.4 Narrative review

Following data synthesis, a narrative review approach was used to examine the findings in the context of evidence-based diagnosis and management, and contemporary approaches to understanding disease causes and experiences. Narrative reviews allow researchers to go beyond describing what is known on a topic and provide an interpretation and critique [[6], [7], [8]].

## 3 Results

### 3.1 Data sources

**Fig. 1:**
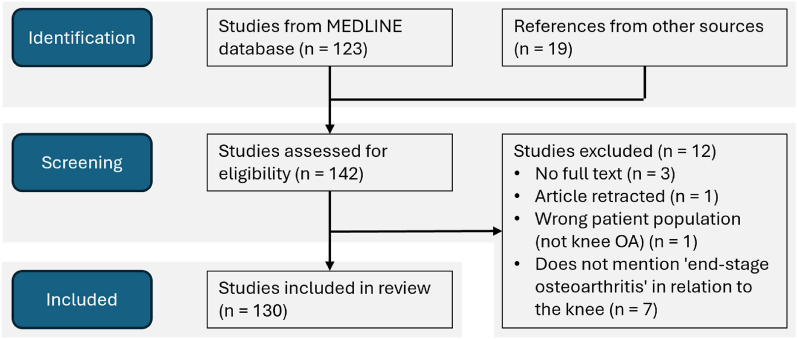
PRISMA flow diagram of number and source of articles/documents screened and included in the review.

The search yielded 142 articles. Searching for a specific term meant that almost all identified articles were eligible and the total number of articles/documents included in the review was 130. Fig. 1 shows the number and source of articles/documents screened, excluded and included.

### 3.2 Data summary

Aggregated characteristics of data sources are provided in Table 1. Most of the sources were clinical trials or observational studies (60%), from countries where the dominant language is not English (64%) and were research articles on the topic of knee surgery (87%).

**Table 1:** Characteristics of data sources.

**Type of study:**	n (%)
Randomised or non-randomised clinical trial (two group comparison study)	53 (41)
Systematic, scoping, rapid or narrative review	35 (27)
Observational study (one group or population study)	25 (19)
Clinical practice guideline	3 (2)
Other (e.g., commentary, opinion)	14 (11)
**Country of study:**	**n (%)**
United States	21 (16)
Australia	13 (10)
UK	11 (8)
Canada	2 (2)
Other (non-English speaking excl China)	54 (42)
China	29 (22)
**Profession/organisation leading the study:**	**n (%)**
Surgeons or department of surgery	83 (64)
Physiotherapists, physical therapists or physiotherapy department	17 (13)
Other medical, e.g., general practitioners, primary care, rheumatologists/y	18 (14)
Exercise physiologists, exercise service	1 (1)
Rehabilitation service	1 (1)
Other (e.g., advocacy, government, engineers, biomechanists)	10 (8)
**Articles related to surgery** (e.g., pre-, during or post- surgery care)**:**	**n (%)**
Yes	113 (87)
No	17 (13)

### 3.3 Definitions of end-stage knee osteoarthritis (KOA)

Twenty (15%) of the articles mentioning end-stage knee OA provided any sort of definition for the term. All text extracted from these 20 articles is provided in Appendix B.

The thematic analysis identified four main approaches to defining end-stage knee OA:

1. **Radiographic evidence approaches.** The most common approach to defining end-stage knee OA was based on *radiographic evidence.* The majority of these definitions relied on the Kellgren-Lawrence (KL) grading system, with KL Grade 4 being the primary marker of end-stage OA [[12], [13], [14], [15], [16], [17], [18]]. Some definitions also specified multiple compartments being affected (according to radiographic evidence) [19,20], such as “KL ≥ 3 in at least two compartments” (Meert, 2023). Some articles provided specific radiographic features that constitute the end-stage [19,[21], [22], [23], [24], [25]]. These descriptive radiological features included “bone-on-bone”, “severe narrowing”, “full-thickness cartilage loss” or “bony deformity”, (which in turn should be defined but were not).
2. **Clinical criteria or symptom burden approaches.** A few definitions were based on clinical symptoms or functional limitations [26,27], although this was less common, were often used in combination with radiographic evidence, and the clinical elements lacked specificity and detail. For example, “no or minimal joint space with inability to cope with pain” [26].
3. **Surgical indication approach.** Some definitions equated end-stage OA with being a candidate for total knee arthroplasty (TKA) [19,25,28].
4. **Integrated radiographic and clinical algorithm.** This approach incorporated both radiographically-assessed knee structure and symptom burden. What sets this approach apart from the other definitions was that it integrated the multiple factors into a *decision rule* [29]. The clinical symptoms were clearly specified (pain severity, disability, frequency of knee pain) and measured using validated tools with quantified thresholds. The algorithm attempted to accommodate for the spectrum of clinical realities and patient experiences where severe symptoms can qualify as ‘end-stage’ even if radiographic OA was mild/moderate. This potentially aligns better with patient-centred care where symptoms should be the main driver of clinical decisions.

### 3.4 Clinical applications of end-stage KOA

The vast majority of included sources agreed that TKA is the best and most common treatment for end-stage knee OA (Appendix C). TKA was frequently described as the “gold standard,” “treatment of choice,” or the “standard” surgical treatment for end-stage OA. It was reported to be effective in reducing pain, improving function, and enhancing quality of life (QOL).

Some studies discussed specific conservative treatment options for end-stage knee OA including physiotherapy programs [30], bariatric surgery [31], joint distraction [32] and drugs [33]. While none claimed conservative treatments were able to fully resolve the condition, they suggested improvement was possible, and in some cases that surgery could be delayed or avoided. Several sources said surgery was required when people had end-stage OA *and* all conservative treatments had “failed”. Neither a trial of conservative treatment, nor failure, were clearly described. One source claimed that the annual rate of surgery for people with end-stage OA was only 1.6–11.9% [34] raising concerns about the accuracy of using of the term interchangeably with the need for surgery.

Studies identified several factors that were *associated with* end-stage knee OA (but not necessarily causing or caused by it), for example, inability to exercise [13], muscle weakness [13,35,36], insufficient anterior cruciate ligament [37], flexion contracture [38], reduced standing balance with increased falls risk [14,38], reduced physical functioning [14,18], and bony deformity [39].

## 4 Discussion and critique

The primary aim of this scoping and narrative review was to explore the definitions and usages of the term ‘end-stage knee OA’ in order to provide conceptual clarity and support future research and clinical practice. Our findings revealed a somewhat discordant landscape in the published literature. The term is highly prevalent, appearing frequently in articles, particularly those related to surgical management of knee OA, and from both English-speaking and non-English-speaking countries. However, most articles using the term failed to provide a definition, leaving its meaning open to interpretation. Those articles providing a definition mostly used radiographic evidence, or sometimes combined radiographic findings with an ill-defined clinical element such as “inability to cope with pain” [26]. Many articles used the term interchangeably with ‘need for surgery’. One article defined end-stage knee OA by integrating the radiographic and clinical elements into a clear and implementable decision rule [29].

Our thematic analysis findings revealed concerns with how end-stage knee OA is defined in the literature and used in clinical practice. Firstly, while the term is in frequent use, it is rarely defined (only 15% of articles). A definition was often missing even when the term was used as a reason for surgery or as a participant inclusion criterion. Within the small number of definitions found, there was variability. Some definitions were solely based on radiographic evidence; others incorporated clinical features or perceived need for surgery. Within the radiographic evidence approach, there was inconsistent use of KL Grade 3, with some definitions including it, and others excluding this grade. Inconsistent inclusion of KL Grade 3 risks diluting the concept. Furthermore, KL Grade only refers to the tibiofemoral compartment and therefore omits the role of the patellofemoral compartment in the presentation.

When clinical features were incorporated, they typically lacked specificity and detail (e.g., “inability to cope with pain” [26]). End-stage knee OA was sometimes used as a proxy for surgical eligibility, but surgical eligibility is also used as the definition of end-stage OA. This is problematic because it creates a circular argument whereby end-stage OA is the indication for surgery at the same time as a need for surgery being used as the definition of end-stage OA. End-stage OA and need for surgery are not necessarily the same thing [34,40] and should not be used interchangeably. Finally, given that the majority of articles relating to surgery stated that end-stage OA was the reason for surgery, the lack of a clear definition including the multiple factors that should be considered in this decision; including symptom, function, biomechanical, psychosocial, and comorbidity factors, is concerning. One author commented: “The decision whether to undergo joint replacement for patients with knee OA is often difficult, with no clear-cut indications for the procedure. The reasons for this failure may be, as least in part, due to the complexity and diversity of end-stage knee OA” [41].

In addition to the identified problems with defining end-stage knee OA, our findings also highlight a disconnect between the term and actual clinician and patient experiences. Our findings, and research evidence, e.g., Refs. [40,42,43], indicate that it is not always the case that having end-stage knee OA means a limit has been reached and the condition is irreversible. Our review found several instances where research participants considered to have end-stage knee OA experienced improvements through non-surgical interventions, or no longer required surgery during the follow-up period [42,43]. The reversibility is not necessarily a flaw of a definition, or a decision rule, used to define this construct, but rather the problem is with the words used. Research articles may refer to a “change in end-stage knee OA” [44], which sounds like an oxymoron. We contend that the word ‘end-stage’ may understood by many people to mean a limit has been reached and there is no point persevering with that knee. While there is evidence the term should not be defined or used to mean irreversible, we also argue that because of how ‘end-stage’ might be interpreted, the term should be avoided altogether, and replaced by alternatives that better reflect the reality.

Furthermore, the condition can still worsen after being labelled end-stage, and symptoms can fluctuate over time, both in the short and long term, in this group. The term therefore fails to capture the variability in patient experiences and the potential for improvement or management without surgical intervention. Some literature refers to ‘severe’ end-stage knee OA or ‘symptomatic’ end-stage disease, implying that end-stage knee OA can be non-severe or asymptomatic. This incongruity undermines the term’s utility in clinical communications. Additionally, the heavy reliance on radiographic imaging for defining end-stage is problematic, given that radiographic changes are known to be unreliable predictors of symptoms and function [45,46], which should be the primary indictor of need for surgery. Recent advances in our knowledge of OA, including the role of inflammation, metabolic factors, and psychosocial aspects in the pain experience, challenge the simplistic notion of an ‘end stage’ in this condition. Moreover, growing evidence suggests that surgery is not the only option at any stage of OA, emphasising the importance of individualised, patient-centred approaches to management decisions.

Accumulating knowledge of the impact that words can have on people’s expectations and behaviours, raises further concerns about the term’s appropriateness and its potential impact on clinical decision-making and patient choices. The power of words should not be underestimated in the context of patient care [47]. We suggest that the term end-stage knee OA may evoke a sense of hopelessness or inevitability in patients, potentially biasing them towards surgical options and discouraging exploration of non-surgical management strategies. This is particularly concerning given that many patients presenting for surgery have not fully explored or adhered to non-surgical options [[48], [49], [50]]. We argue that giving someone the label of having end-stage knee OA could be harmful for some people who prefer not to have surgery or cannot have surgery. The label may also influence healthcare providers, potentially leading them to have a bias towards recommending surgical interventions.

The use of more patient-centred language that focuses on individual experiences and values, rather than fatalistic, pessimistic terms, may help patients maintain hope and engagement in self-management [51]. A different choice of words could encourage the ongoing use of non-surgical strategies, even when surgery is being considered, and support more personalised decision-making processes. By moving away from the term ‘end-stage’, we may be able to create a more nuanced and accurate dialogue about knee OA management, one that better reflects the complex, individual and variable nature of the condition and empowers patients to explore all available options for improving their QOL.

The review findings have led us to formulate two recommendations:

1. Use the term ‘surgical threshold’ to describe the stage when the person is eligible for considering surgery. Determining the surgical threshold should involve consideration of multidimensional criteria (radiographic findings, symptoms, function, treatment history, impact and other social and contextual factors). Symptoms and function should be measured using validated scales (e.g., pain severity numeric rating scale [52], WOMAC [52,53]). Further, to this recommendation, clinicians should use the term ‘surgical threshold’ in a way that guides patients and other clinicians with decision-making in a clear and more accurate way. That is, the patient has reached a point when surgery becomes an *option* for consideration. Text Box 1 provides a suggestion on how a conversation about the option of surgery could be worded avoiding the term end-stage. We believe this kind of framing will help by centring the decision-making on the patient’s needs, lived experience and values, emphasising that imaging does not dictate treatment, and keeping the door open for both surgical and non-surgical management being part of the care plan over the long term.
  Text box 1 **Suggested wording where ‘surgical threshold’ replaces the term end-stage knee osteoarthritis in a clinical conversation.**
*It sounds like your osteoarthritis is at a point where it’s really limiting your daily life and causing you significant pain. We can call this a ‘surgical threshold’ – which means surgery could now be one of the options on the table, not because of how your joint looks on an x-ray, or because someone else said you should have surgery, but because your symptoms are no longer manageable in the way you are able to tolerate - and you are the one living with these symptoms. It’s important to know that this doesn’t mean you have to have surgery. Some people do choose it when their pain and limitations get too much for them, however others still find ways to manage without surgery. Either way, we always have other options to support your pain, mobility, and QOL - through physical activities, exercise programs, medications, lifestyle changes and techniques for managing your thinking and mood. Ultimately, the decision is yours. You need to choose what feels right for you - not just based on the look of the joint, but based on your goals, values, and everything else that’s going on with your health and in your day-to-day life.*
  Alt-text: Text box 1

2. In a research context, when radiographic findings are the important criteria or endpoint, the recommended alternative to end-stage knee OA would be to use the term ‘advanced radiographic knee OA’ or simply ‘KL Grade 4’. Similarly, in studies when the outcome of interest is pain or function, the level of pain and/or function should be clearly stated and ideally specified. For example, KOA ‘with severe functional limitation’ or ‘with pain greater than 6/10’. A more complex algorithm or decision rule that combines pain, function and radiographic findings, such as that developed Driban and colleagues [29,40,44,54] (see Theme 4 from our data analysis of definitions) might be the most appropriate option for either participant selection into studies on surgical interventions, or as a binary end-point or continuous outcome in clinical trials. The algorithm has been shown to be valid, reproducible, avoids the pitfalls of non-medical, sociological factors determining surgical rates, and mimics clinical practice [29,44,54]. However, rather than name this construct end-stage knee OA (or esKOA [29]), preferable terms would be “advanced” or “severe” knee OA.

### 4.1 Limitations

This scoping and narrative review has several limitations to consider. The aim was to explore and discuss the concept definitions and variations in usage across the literature rather than to quantify how many times the term appears. The literature including the term “end-stage knee OA” is large but rather homogeneous, with many brief mentions and similar usages. Retrieving a larger number of articles was not warranted as it would yield limited additional conceptual insight. Purposeful, stratified sampling with pre-specified limits (date and article type) allowed us to efficiently capture the range of definitions and contexts.

We did not search for closely associated terms such as ‘end-stage arthritis’, and ‘end-stage symptomatic OA’, or synonyms such as ‘osteoarthrosis’, ‘arthrosis’ or ‘gonarthrosis’. However, our search methodology enabled us to capture a comprehensive overview of how the term end-stage knee OA is used in the literature, and allowed us to gain an accurate understanding of how the term is defined and used. Our subsequent narrative review allowed us to highlight problems including inconsistencies, ambiguities and inaccuracies. Narrative reviews allow reviewers to develop a deeper understanding and interpretation of the topic, but are more open to bias [7,8]. Our critique is informed by current evidence, but we acknowledge that our interpretation may differ from others. Future research could build on these findings with more targeted primary research to address specific questions arising from this scoping review and greater involvement of a range of stakeholders in the interpretation.

## 5 Conclusion

This scoping and narrative review of the term end-stage knee OA revealed significant gaps and inconsistencies in its definition and application. Despite its widespread use, we found a notable lack of consensus on what is meant by ‘end-stage’, with most literature failing to provide a clear definition. The lack of a universally agreed-upon definition, coupled with its frequent use as a justification for surgery, could be creating confusion and potentially contributing to inappropriate patient management and difficulty interpreting research findings.

Furthermore, we highlighted the problems with the term due to the power of language in shaping patient expectations and management decisions. The term ‘end-stage’ may inadvertently create a sense of hopelessness or inevitability, potentially biasing both patients and clinicians towards surgical interventions and discouragingv exploration of non-surgical management strategies. We therefore proposed two recommendations. Firstly, using the term ‘surgical threshold’ to indicate when surgery becomes an option to consider. Secondly, when radiological findings are the key concern, the term ‘advanced radiographic knee OA’, or simply ‘KL Grade 4’, would be preferable. Ultimately, by moving away from the potentially limiting concept of end-stage knee OA, we may better serve the needs of patients living with this condition.

## Author contributions

Thorlene Egerton: Conceptualisation, study design, data collection and analysis, interpretation of findings, writing/editing of the original paper, final approval of submitted version. Penny Campbell: data collection and analysis, manuscript critical review/editing for important intellectual content, and final approval of submitted version. Thorlene Egerton (thorlene.egerton@unimelb.edu.au) takes responsibility for the integrity of the work as a whole, from inception to finished article.

## Role of funding sources

Not applicable.

## Conflict of interest

The authors have no conflicts of interest to declare.

## Figures and Tables

**Table:** 

Step	Terms/filters	Hits
1	(“End-stage knee osteoarthritis” or “end stage knee osteoarthritis” or “end-stage knee OA” or “end stage knee OA” or “end-stage osteoarthritis” or “end stage osteoarthritis” or “end-stage OA” or “end stage OA” or “endstage osteoarthritis” or “endstage OA” or “endstage knee osteoarthritis” or “endstage knee OA”).mp. [mp = title, book title, abstract, original title, name of substance word, subject heading word, floating sub-heading word, keyword heading word, organism supplementary concept word, protocol supplementary concept word, rare disease supplementary concept word, unique identifier, synonyms, population supplementary concept word, anatomy supplementary concept word]	1061
2	Limit 1 to (English language and humans and yr = "2015 -current")	595
3	knee.mp. [mp = title, book title, abstract, original title, name of substance word, subject heading word, floating sub-heading word, keyword heading word, organism supplementary concept word, protocol supplementary concept word, rare disease supplementary concept word, unique identifier, synonyms, population supplementary concept word, anatomy supplementary concept word]	227,122
4	2 and 3	467
5	Limit 4 to (clinical trial protocol or clinical trial or guideline or observational study or practice guideline or randomised controlled trial or “review” or “scoping review” or “systematic review”)	**123**

**Table:** 

Organisation	What was searched	What was found
NICE	4 relevant guidelines searched	4 included resources
OARSI	All documents including guidelines and articles in a special edition of O&C (2015)	2 reports and 1 article were included
ACR	Searched across the website including clinical tools, guidelines and patient resources	No mention of end-stage knee OA
European league against rheumatism (EULAR)	Searched website and annals of rheumatic diseases pages	Three full text articles identified and included.
American academy of orthopaedic surgeons (AAOS)	Searched across the website (aaos.org) including guidelines, and ‘OrthoInfo’ patient facing pages. The AAOS now articles were for members only.	No mention.
Arthritis research UK (versus)	All available online resources/articles	No mention of end-stage OA
RACGP	All available online resources/articles	Three full text articles identified and included
Arthritis foundation	All available online resources/articles	Mentioned in the following: −1 × event/publication −2 × news articles −1 × general info page
BSR	All available online resources/articles	No mention of end-stage OA
World health organisation (WHO)	Used search function to identify documents and searched quite a few of them	No mentions
Canadian rheumatology association (CRA)	Searched main landing page and looked through guidelines for ‘osteoarthritis’ (rheum.ca)	No mention (focus is on RA)

Up to date	Unable to search (subscription required)	Not applicable

APTA	Unable to search (members only)	Not applicable

NHS	All available online resources/articles	No mentions
American association of hip and knee surgeons (AAHKS)	Searched main site and its meeting/uploads pages (www.aahks.org and https://hipkneeinfo.org/)	1. Association of Hip and Knee Surgeons blog/response page — “AAHKS response to Total knee replacement study” (https://www.aahks.org/tkr/) 2. Association of Hip and Knee Surgeons comment letter (PDF) — “AAHKS - 2024 MA UM information collection request comment letter” (AAHKS upload) (https://www.aahks.org/wp-content/uploads/2024/11/AAHKS-2024-MA-UM-Information-Collection-Request-Comment-Letter.pdf)
Arthritis Australia	Searched all pages of website arthritisaustralia.com.au, and patient booklet “taking control of your osteoarthritis” and MyJointPain site	No mentions
Osteoarthritis foundation international (OAFI)	Searched all pages on the website (https://www.oafifoundation.com/) including white papers and patient facing resources	No mentions
Arthritis society (Canada)	Searched all pages on the website of arthritis.ca	No mentions

**Table:** 

#	Author (Year)	Definition provided∗
134	Castagno (2025)	A KL grade of 4 was used to identify patients with radiographic end-stage
131	American association of hip and knee surgeons (AAHKS) (2024)	Near end-stage to end-stage osteoarthritis (primary or secondary) (i.e., near or complete loss of joint space, bone-on-bone with or without varus, or valgus malalignment). Radiological evidence of end stage joint disease (=bone-on-bone articulation)
111	Wang (2018)	The medial, lateral, patellofemoral compartment was given an individual Kellgren lawrence grade on the radiographs, and those graded 4 were defined as end-stage OA.
100	Sugawara (2017)	… radiographic end-stage knee OA classified as Kellgren and lawrence grade 4
90	Ren (2025)	End-stage knee osteoarthritis, classified as Kellgren-lawrence stage IV
91	Salis (2024)	End-stage knee osteoarthritis (KOA) was defined if a knee meets one of two criteria: (1) moderate, intense, or severe symptoms of KOA indicated by pain and disability measurement and severe KOA indicated by radiographically-assessed knee structure; (2) intense or severe symptoms of KOA indicated by pain and disability measurement and frequent knee pain with mild or moderate KOA as indicated by radiographically-assessed knee structure.
95	Sideris (2022)	Enrolment criteria included adult patients undergoing unilateral TKA for severe end-stage OA. OA was deemed severe [end-stage] by radiologist or surgeon using descriptors of “severe narrowing” and/or “bone on bone”.
84	Palmer (2019)	End-stage osteoarthritis [is] full-thickness cartilage loss (>1 cm) or bony deformity (Kellgren-Lawrence grade 4).
81	Nicholls (2019)	… End-stage OA (i.e., KL grade 4)
75	Meessen (2020)	End-stage knee/hip OA (TJA) was defined as indication for arthroplasty surgery.
74	Meert (2023)	Enrolment criteria included adult patients undergoing unilateral TKA for severe end-stage OA. OA was deemed severe [end-stage] by radiologist or surgeon using descriptors of “severe narrowing” and/or “bone on bone”. End-stage knee OA in at least 2 compartments (i.e., KL score >3)
72	Madry (2016)	All patients had severe [end-stage] knee OA (i.e., Kellgren and lawrence grade 4) that was refractory to previous treatments.
68	Liu (2017)	… End-stage knee OA (i.e., K/L grade 4)
63	Li (2019)	… clinical and radiographic evidence diagnosed with end-stage knee OA according to the diagnosis criteria. [No info on how to diagnose end-stage OA or diagnostic criteria in cited article].
60	Lee (2022)	All participants met the radiographic criteria for end-stage knee OA: Kellgren lawrence (K-L) grade 3 and grade 4, on at least 1 side. The K-L grading system, accepted by world health organisation in 1961, is the most commonly used knee OA severity grading system. The system classifies knee OA severity into 5 grades: Grade 0 to grade 4. The criteria for each grade are: Grade 3 = multiple osteophytes, definite joint space narrowing, sclerosis. Grade 4 = large osteophytes, marked joint space narrowing, severe sclerosis
56	Kuang (2017)	… Degeneration of articular cartilage
39	Giannini (2015)	End-stage post-traumatic knee arthritis [according to the Kellgren Lawrence grading system].
30	Duong (2023)	… Defined as no or minimal joint space with inability to cope with pain
19	Cherian (2015)	Kellgren Lawrence grade 3 and 4
15	Cakmak (2025)	Kellgren-Lawrence grade 4 knee osteoarthritis
10	Beckers (2019)	… Bone on bone

∗Quotes from articles edited slightly for readability.

## Appendix A Search strategy

Medline (OVID)

Ovid MEDLINE(R) ALL <1946 to August 11, 2025>

Websites of 18 key English-language organisations and clinical practice guidelines related to knee osteoarthritis (OA) were hand searched. Two were excluded as behind a paywall. The search yielded resulting in 19 additional resources:
