# Supplementary material for: What is end-stage knee osteoarthritis? A scoping and narrative review
Source: Osteoarthr Cartil Open. 2026 Apr 30;8(2):100808. doi: 10.1016/j.ocarto.2026.100808 (PMC13196067; doi:10.1016/j.ocarto.2026.100808)
Supplement: Multimedia component 1 [file mmc1.docx]

# **Appendix C. Extracted clinical practice recommendations related to end-stage knee osteoarthritis, and other discussion of end-stage knee osteoarthritis**

| **#** | **Author (Year)** | **CLINICAL PRACTICE use*** | **OTHER discussion of relevance to end-stage (knee) OA*** |
| --- | --- | --- | --- |
| 2 | Aljehani (2021) | It is the assumption that those individuals on a waiting list for TKA have end-stage knee OA |  |
| 3 | An (2021) | The results of this study demonstrate the early benefits of a [physical therapy] program in elderly female patients with end-stage osteoarthritis |  |
| 4 | An (2023) | Total knee arthroplasty (TKA) is an effective treatment for end-stage osteoarthritis. |  |
| 5 | Anderson (2022) | Total knee replacement (TKR) is a common operation typically performed for end-stage knee osteoarthritis. |  |
| 6 | Andrade (2022) | Total knee arthroplasty is used to treat end-stage knee osteoarthritis with great results. |  |
| 7 | Assiotis (2019) | Total knee arthroplasty is a common operation for treating patients with end-stage knee osteoarthritis and generally has a  good outcome. |  |
| 9 | Beckers (2024) | In patients with osteopetrosis with end-stage OA, [total joint arthroplasty] is an effective treatment with good outcomes. |  |
| 10 | Beckers (2019) | Medial [unilateral knee arthroplasty] has been increasingly used in the treatment of end-stage anteromedial OA with good clinical results and  accompanying outcome. |  |
| 12 | Blikman (2022) | The foremost insight is that end-stage knee OA patients still possess the ability to respond well to conservative duloxetine treatment.  Adding duloxetine treatment to usual care seems to be especially beneficial for end-stage knee OA patients with neuropathic-like symptoms or central sensitization. |  |
| 13 | Bove (2022) | The most effective treatment for end-stage knee OA is total knee arthroplasty (TKA) surgery |  |
| 16 | Calatayud (2017) | knee replacement surgery is the most common and effective treatment to reduce pain and improve functionality [in end-stage osteoarthritis] | Two patients in the intervention group (high intensity pre-operative exercise) [who had end-stage osteoarthritis] withdrew from the study because they decided not to have surgery. |
| 19 | Cherian (2015) | ...many patients [with end-stage osteoarthritis] often require joint arthroplasty.  One of the challenges of treating patients with painful end-stage osteoarthritis is dealing with their common quadriceps and hamstring muscle weakness and their inability to tolerate exercises | ...the natural history of osteoarthritis [is for it to] progress to end-stage degenerative joint disease.  [Some patients were able to achieve] functional improvements in patients who had end-stage knee osteoarthritis. |
| 20 | Choong (2021) | Total joint arthroplasty (TJA) is an effective treatment for end-stage osteoarthritis |  |
| 21 | Clement (2022) | Total knee replacement (TKR) is a cost-effective intervention for the management of end-stage OA of the knee |  |
| 22 | Cochrane (2021) | Total knee arthroplasty is the standard surgical treatment for end-stage osteoarthritis |  |
| 24 | Cools (2023) | Total knee arthroplasty (TKA) is a well-known surgical procedure performed to address end stage osteoarthritis. | ...the ACL integrity in terms of strength and proprioception may be questionable in cases of end-stage osteoarthritis. |
| 26 | Dowsey (2022) |  | [Total knee arthroplasty] was declined by 12 participants (29.3%) in the intervention group [who had barriatric surgery to lose weight] because of symptom improvement, whereas 2 participants (4.9%) in the [treatment as usual] group declined TKA. |
| 27 | Dowsey (2016) | Total knee arthroplasty (TKA) is a clinically effective treatment for people with end-stage knee OA |  |
| 28 | Dragosloveanu (2023) | Total knee arthroplasties (TKAs) are the most effective surgical treatment for end-stage knee osteoarthritis |  |
| 29 | Drummer (2022) |  | some patients with end-stage OA possess greater dysregulated skeletal muscle surrounding the diseased joint. |
| 30 | Duong (2023) | Surgical referral for knee joint replacement can be considered for patients with end-stage OA  Total knee replacement is indicated when patients have used all appropriate first- and second-line conservative treatment options and have end-stage OA | The indication for surgical interventions is typically end-stage knee OA (defined as no or minimal joint space with inability to cope with pain) after conservative options have not effectively relieved symptoms. |
| 32 | ElkjaerChristensen (2024) | nearly 50% of end-stage OA patients [are] eligible for mUKA [medial unicompartmental knee arthroplasty] [as opposed to total knee arthroplasty] |  |
| 34 | Evans (2019) | Knee replacements are the mainstay of treatment for end-stage osteoarthritis and are effective |  |
| 35 | Fan (2018) | Total knee arthroplasty (TKA) is a best choice for the treatment of end-stage osteoarthritis due to degeneration of articular cartilage |  |
| 45 | Harato (2017) | total knee arthroplasty (TKA) has traditionally been performed as an effective treatment for patients with end-stage knee OA, by relieving pain, restoring function, and correcting deformity. | flexion contracture is frequently seen in patients with end-stage knee OA.  It has been demonstrated that standing balance is exacerbated in patients with end-stage knee OA, and these facts would lead to an increased risk of falling in such patients.  Patients with end-stage medial knee OA in the current study had balance impairment during [one leg stand]. |
| 48 | Husted (2018) |  | Clinically, patients diagnosed with end-stage knee OA who are awaiting TKA to reduce pain and disability are reported to have 35% reduced knee-extensor strength compared to healthy, age-matched persons |
| 49 | Ibrahim (2017) |  | A patient-centered counseling and educational intervention may help to address racial variations in the use of TKR for the management of end-stage OA of the knee. |
| 50 | Jansen (2022) |  | Over the past 30 years, joint distraction has emerged as a joint-preserving treatment for patients with end-stage OA who are being considered for joint replacement surgery, with a gradually growing promise for implementation in regular clinical practice. |
| 53 | Krampe (2023) | Total knee arthroplasty is performed to relieve knee pain and disability related to end-stage osteoarthritis. |  |
| 55 | Kris (2025) | Total knee arthroplasty (TKA) is a common surgical intervention for end-stage knee osteoarthritis, yet up to 20 % of patients report dissatisfaction with surgery. |  |
| 56 | Kuang (2017) | Total knee arthroplasty (TKA) is gradually emerging as the treatment of choice for end-stage osteoarthritis. |  |
| 58 | Larsen (2020) | Total knee arthroplasty (TKA) is considered an effective treatment for pain relief and improved physical performances in end-stage knee osteoarthritis.  End-stage knee OA is often treated with a knee replacement. |  |
| 59 | Lee (2021) | Total knee arthroplasty (TKA) is used to treat end-stage osteoarthritis.  Total knee arthroplasty (TKA) is used to treat patients with end-stage osteoarthritis who experience pain and struggle to perform activities of daily living owing to having degenerative knee joints. |  |
| 60 | Lee (2022) | ...a preventive approach to managing osteosarcopenia might be warranted in those with end-stage knee OA who are scheduled to undergo primary TKA.  ...measurement of osteoporosis and appendicular skeletal lean mass will be helpful for assessing the physical function of frail patients with end-stage knee OA. | The results presented herein suggest that, when compared with age-matched people, patients with end-stage knee OA have reduced physical function in important areas of daily activity such as maintaining gait speed and balance. |
| 64 | Li (2019) | Total knee arthroplasty (TKA) is an optimal option for patients with middle-to-end-stage knee osteoarthritis. |  |
| 66 | Lin (2025) | Total knee arthroplasty (TKA) is the gold-standard treatment for end-stage osteoarthritis (OA).  Patients with end-stage knee osteoarthritis (KOA) typically undergo TKA as the standard treatment to restore joint function and alleviate pain. |  |
| 67 | Lin (2019) | Total knee arthroplasty (TKA) is the most common treatment for end-stage knee osteoarthritis.  TKA is commonly performed to relieve joint pain, and to improve quality of life in patients who have end-stage osteoarthritis. |  |
| 68 | Liu (2017) |  | The present study also showed that the osteophyte formation evaluated by MRI was associated with disability in daily living in patients with end-stage knee OA, suggesting a causal relationship between osteophyte formation and disability in daily living in patients with end-stage knee OA who were considering whether to undergo TKA.  ...disability of daily living was suggested to be one of the important factors for undergoing TKA in patients with end-stage knee OA. |
| 69 | Liu (2022) | Total knee arthroplasty (TKA) is effective in relieving pain and improving function in patients with end-stage knee osteoarthritis. |  |
| 70 | Londhe (2025) | Total knee arthroplasty (TKA) is crucial for alleviating pain and improving the quality of life in patients with end-stage knee arthritis. |  |
| 71 | Madry (2022) | Total joint arthroplasty (TJA) represents the only valuable, established surgical option for severe, end stage OA. |  |
| 76 | Men (2021) |  | Patients with end-stage osteoarthritis of the knee may also have developmental joint deformities such as bunion deformity, subtrochanteric deformity, and forefoot and hindfoot deformities |
| 83 | Padhya (2024) | Hip and knee arthroplasty surgeries are the gold standard for the treatment of end-stage osteoarthritis.  Hip and knee arthroplasty surgeries are essential for treating end-stage osteoarthritis, providing significant functional improvements. |  |
| 84 | Palmer (2019) | End-stage osteoarthritis of the knee is successfully treated with a knee replacement.  End-stage, "bone-on-bone osteoarthritis" is treated dependably with arthroplasty in the form of total or unicompartmental knee replacement. |  |
| 89 | Rantasalo (2018) | Total knee arthroplasty (TKA) is a common and highly effective orthopaedic procedure for treating end-stage knee osteoarthritis  with good long-term results when conservative treatment provides inadequate relief. |  |
| 90 | Ren (2025) | Total knee arthroplasty (TKA) remains the primary treatment for end-stage knee osteoarthritis, with surgical navigation robots showing significant clinical benefits. |  |
| 92 | Schache (2016) | Total knee arthroplasty (TKA) is effective in reducing pain and improving function for end-stage knee osteoarthritis.  Total knee arthoplasty (TKA) leads to significant improvements in pain and the performance of functional activities such as walking for patients with end-stage knee OA. | Prior to surgery, patients with end-stage knee OA demonstrate reduced hip abductor strength. |
| 93 | Sibilska (2020) |  | Nowadays, SONK [spontaneous osteonecrosis of the knee] is said to be a relatively common disease usually described as a focal, subchondral lesion which may lead to an end-stage osteoarthritis of the knee. |
| 94 | Sicat (2021) | Total knee arthroplasty (TKA) is the gold standard for treatment of end-stage osteoarthritis. |  |
| 99 | Strahl (2022) | When conservative management fails, total joint arthroplasty (TJA) is the treatment of choice for end-stage OA. |  |
| 100 | Sugawara (2017) |  | The decision whether to undergo joint replacement for patients with knee OA is often difficult, with no clear-cut indications for the procedure. The reasons for this failure may be, as least in part, due to the complexity and diversity of end-stage knee OA. |
| 101 | (Suhas Masilamani) 2025 | Total knee arthroplasty (TKA) significantly alleviates pain and improves functionality in patients with end-stage knee osteoarthritis. | End-stage osteoarthritis (OA) of the hip and knee joints can be very debilitating for patients, with a significant reduction in the quality of life (QOL) and difficulty with activities of daily living (ADL) |
| 102 | (Teissier) 2020 | Total knee arthroplasty (TKA) is the preferred surgical treatment of end stage osteoarthritis of the knee. |  |
| 103 | Temporiti (2022) | Total knee arthroplasty (TKA) represents an effective and definitive treatment for end-stage knee osteoarthritis, able to relieve pain, improve functional level, and quality of life. |  |
| 104 | Teng (2020) | Total knee arthroplasty (TKA) is gradually emerging as the treatment of choice for end-stage osteoarthritis.  Total knee arthroplasty (TKA) is a successful surgical procedure as the treatment of choice for end-stage osteoarthritis which can improve quality of life and functional for patients. |  |
| 105 | Terradas-Monllor (2021) | Knee arthroplasty (KA) is an effective and cost-effective treatment for end-stage knee osteoarthritis.   Orthopedic surgeries such as KA are considered for end-stage knee osteoarthritis when conservative treatment has failed. |  |
| 106 | van der Woude (2017) | In end-stage osteoarthritis (OA) of the knee a total knee arthroplasty (TKA) is generally indicated. |  |
| 107 | Verra (2016) | A total knee arthroplasty (TKA) is generally accepted to be an effective surgical treatment for end-stage knee OA. | End-stage knee osteoarthritis (OA) results in total knee arthroplasty (TKA) surgery. |
| 109 | Wang (2018) |  | Patients with end-stage OA displayed more severe synovitis compared to patients who did not have an indication for a knee arthroplasty. |
| 110 | Wang (2024) | For end-stage knee OA patients, total knee replacement surgery is one of the most effective surgical interventions for pain relief and functional recovery.  Those with end-stage knee OA would experience increased discomfort, which could result in reduced mobility, an increased risk of sarcopenia, and a vitamin D deficiency that needs to be addressed.  Patients with end-stage knee OA will be recommended to take the TKR as the viable option after the failure of conservative, non-surgical management. | The number of people with end-stage knee OA who are waiting for surgery is anticipated to rise sharply as the population age.  Patients with end-stage knee OA frequently give up on physical activity to prevent joint stiffness and pain, which can negatively impact overall muscle health and localized knee function. |
| 111 | Wang (2018) | Surgical intervention, such as knee arthroplasty, can be used in those with end-stage knee OA.  Total knee arthroplasty (TKA) is one of the most common surgical procedures for end-stage knee OA, with promising clinical outcomes.  Total knee arthroplasty (TKA) is the most successful treatment for end-stage knee osteoarthritis (OA), offering patients significant symptomatic relief and functional improvement, while demonstrating a low long-term failure rate. |  |
| 115 | Yang (2023) | Total knee arthroplasty (TKA) is a surgical procedure primarily used to treat patients with end-stage knee osteoarthritis (KOA).   KA is a reliable surgical intervention for end-stage KOA and is recognized as a cost-effective treatment option. |  |
| 116 | Yang (2021) | Total knee arthroplasty (TKA) is a common and highly effective orthopaedic procedure for treating end-stage knee osteoarthritis,  which can reconstruct joint alignment, alleviate pain and improve joint function. |  |
| 118 | Yue (2025) | Total knee arthroplasty (TKA) is a standard treatment for end-stage knee osteoarthritis (KOA). |  |
| 119 | Zhang (2025) | In recent years, Total knee arthroplasty (TKA) for treating of the end-stage arthritis of the knee developed rapidly. |  |
| 120 | Zhang (2024) |  | In patients with end-stage knee OA, the deficit in knee-extensor muscle strength persists and further deteriorates even after 3 months following TKA |
| 121 | Zhang (2017) | Total knee arthroplasty (TKA) is gradually regarded as an effective choice for end-stage osteoarthritis |  |
| 122 | Zhao (2019) | Total Joint Arthroplasty (TJA) is gradually emerging as the treatment of choice for end-stage osteoarthritis. |  |
| 125 | NationalInstituteforHealthandCareExcellence(NICE) (2015) |  |  |
| 128 | McAlindon (2015) |  | Some studies may also use a total knee replacement as an end-point since it is a clinically relevant end-point that is influenced by symptomatic and structural severity. However, these studies will require a large sample size because the annual incidence rates of total knee replacements among individuals with end-stage OA is low (1.6-11.9%). |
| 130 | OsteoarthritisResearchSocietyInternational (2016) | Total joint replacement surgery (TJR) is a treatment for end-stage OA, and is not considered a cure as there are still limitations remaining post-surgery. | We have no known cure or proven strategy for reducing progression from early to end-stage OA. |
| 131 | AmericanAssociationofHipandKneeSurgeons(AAHKS) (2024) | Insurers have a clear financial incentive to mandate an extended period of nonoperative treatment before pursuing surgical intervention, despite prevailing clinical evidence suggesting that many nonoperative modalities fail to effectively control symptoms associated with end-stage degenerative joint disease of the hip and knee.  Insurance companies should provide greater evidence-based rationale for diagnostic criteria, as end-stage OA patients are being funneled into generic treatment algorithms, delaying access to TKA. | Delays to ... TKA due to prior authorization have been demonstrated to increase postoperative complications, 90-day revision rates, chronic disease and disability, prolong limitations to physical activity in patients with end-stage osteoarthritis (OA), and worsen the quality of life and postoperative outcomes of TJA candidates.  While patients with early or mild OA may benefit from conservative management, those with end-stage symptomatic OA experience severe disability that cannot be alleviated by conservative management. |
| 132 | AmericanAssociationofHipandKneeSurgeons(AAHKS) (2014) | A recent study in the Journal of Bone and Joint Surgery found that knee replacement is a cost-effective treatment for patients with end-stage osteoarthritis |  |
| 133 | Bastiaansen-Jenniskens (2012) |  | Primary and secondary OA are different regarding the onset of the disease, but it has never been proven that the end stage is different |
| 135 | Bomer (2015) | There is no effective therapy to reverse or slow down the disease except for joint replacement surgery at the end stage |  |

* Quotes from articles edited slightly for readability.
